# Cost-Effectiveness of Biosimilars vs Leflunomide in Patients With Rheumatoid Arthritis

**DOI:** 10.1001/jamanetworkopen.2024.18800

**Published:** 2024-06-26

**Authors:** Kuan Peng, Shirley C. W. Chan, Yang Wang, Franco W. T. Cheng, Winnie W. Y. Yeung, Yuanshi Jiao, Esther W. Y. Chan, Ian C. K. Wong, Chak-Sing Lau, Xue Li

**Affiliations:** 1 Department of Medicine, School of Clinical Medicine, Li Ka Shing Faculty of Medicine, The University of Hong Kong, Hong Kong SAR, China; 2 Centre for Safe Medication Practice and Research, Department of Pharmacology and Pharmacy, Li Ka Shing Faculty of Medicine, The University of Hong Kong, Hong Kong SAR, China; 3 School of Nursing, Li Ka Shing Faculty of Medicine, The University of Hong Kong, Hong Kong SAR, China; 4 Laboratory of Data Discovery for Health (D^2^4H), Hong Kong Science Park, Hong Kong SAR, China; 5 School of Pharmacy, Aston University, Birmingham, England

## Abstract

**Importance:**

Among patients with rheumatoid arthritis (RA) who had an inadequate response to methotrexate, a treatment sequence initiated with biosimilar disease-modifying antirheumatic drugs (DMARDs) provides better clinical efficacy compared with conventional synthetic DMARDs recommended by current treatment guidelines; but its cost-effectiveness evidence remains unclear.

**Objective:**

To evaluate the cost-effectiveness of the treatment sequence initiated with biosimilar DMARDs after failure with methotrexate vs leflunomide and inform formulary listing decisions.

**Design, Setting, and Participants:**

This economic evaluation’s cost-effectiveness analysis was performed at a Hong Kong public institution using the Markov disease transition model to simulate the lifetime disease progression and cost for patients with RA, using monetary value in 2022. Scenario and sensitivity analyses were performed to test the internal validity of the modeling conclusion. Participants included patients diagnosed with RA from 2000 to 2021 who were retrieved retrospectively from local electronic medical records to generate model input parameters. Statistical analysis was performed from January 2023 to March 2024.

**Interventions:**

The model assesses 3 competing treatment sequences initiated with biosimilar infliximab (CT-P13), biosimilar adalimumab (ABP-501), and leflunomide; all used in combination with methotrexate.

**Main Outcomes and Measures:**

Lifetime health care cost and quality-adjusted life-years (QALYs) of the simulated cohort.

**Results:**

In total, 25 099 patients with RA were identified (mean [SD] age, 56 [17] years; 19 469 [72.7%] women). In the base-case analysis, the lifetime health care cost and QALYs for the treatment sequence initiated with leflunomide were US $154 632 and 14.82 QALYs, respectively; for biosimilar infliximab, they were US $152 326 and 15.35 QALYs, respectively; and for biosimilar adalimumab, they were US $145 419 and 15.55 QALYs, respectively. Both biosimilar sequences presented lower costs and greater QALYs than the leflunomide sequence. In the deterministic sensitivity analysis, the incremental cost-effectiveness ratio (US$/QALY) comparing biosimilar infliximab sequence vs leflunomide sequence and biosimilar adalimumab sequence vs leflunomide sequence ranged from −15 797 to −8615 and −9088 to 10 238, respectively, all below the predefined willingness-to-pay threshold (US $48 555/QALY gain). In the probabilistic sensitivity analysis, the probability of treatment sequence initiated with leflunomide, biosimilar infliximab, and biosmilar adalimumab being cost-effective out of 10 000 iterations was 0%, 9%, and 91%, respectively.

**Conclusions and Relevance:**

In this economic evaluation study, the treatment sequences initiated with biosimilar DMARDs were cost-effective compared with the treatment sequence initiated with leflunomide in managing patients with RA who experienced failure with the initial methotrexate treatment. These results suggest the need to update clinical treatment guidelines for initiating biosimilars immediately after the failure of methotrexate for patients with RA.

## Key Points

**Question:**

Among patients with rheumatoid arthritis who have inadequate response to methotrexate, is the treatment sequence initiated with biosimilar disease-modifying antirheumatic drugs (DMARDs) cost-effective compared with leflunomide?

**Findings:**

In this economic evaluation of 25 099 patients with rheumatoid arthritis, both base-case and sensitivity analyses found that treatment sequences initiated with biosimilar DMARDs were cost-effective compared with leflunomide.

**Meaning:**

These findings suggest the need to update clinical treatment guidelines for initiating biosimilars immediately after the failure of methotrexate for patients with rheumatoid arthritis.

## Teaser

This economic evaluation examines the cost-effectiveness of the rheumatoid arthritis treatment sequence initiated with biosimilar disease-modifying antirheumatic drugs (DMARDs) after failure with methotrexate vs leflunomide.

## Introduction

Rheumatoid arthritis (RA) is a highly prevalent inflammatory joint disease leading to permanent joint destruction and physical and mental impairment.^1^ Methotrexate has long been the anchor drug for treating RA, given its promising clinical efficacy, safety, and modest treatment cost. However, more than 50% of patients with RA would experience failed methotrexate monotherapy due to inadequate response within 1 year.^2^ Current clinical guidelines recommend using several conventional synthetic disease-modifying antirheumatic drugs (csDMARDs, such as leflunomide, cyclosporine, and hydroxychloroquine) in combination with methotrexate for patients with inadequate methotrexate response in the absence of poor prognostic factors.^3,4^ If combination therapy with csDMARDs does not achieve the treatment goal, a combination of biological DMARDs (bDMARDs, such as infliximab, etanercept, and adalimumab) with methotrexate is recommended subsequently. However, recent evidence has suggested that initiating bDMARDs after the failure of methotrexate presents an improved clinical response rate and drug retention rate compared with csDMARDs in both clinical trials and clinical practice.^5,6,7,8^

A major barrier preventing the use of bDMARDs is the substantial drug cost associated with them.^9^ The cost-effectiveness of bDMARDs, indicating the trade-off between increased medication expenditure and the additional health benefit, is vital for public formulary enlisting and reimbursement decisions. Many studies have attempted to elucidate whether initiating bDMARDs after the failure of methotrexate is cost-effective compared with csDMARDs, where the conclusions were controversial and highly sensitive to the price of bDMARDs.^10,11^ Recently approved biosimilars share similar functions and structure to their reference biologics without meaningful clinical differences in efficacy and safety.^12,13^ Fifty-eight biosimilars have been approved in Europe, and the average price reduction ranges from 25% to 55%.^14,15^ Introducing biosimilars could improve the cost-effectiveness of bDMARDs by reducing medication costs. According to the latest public Drug Formulary in Hong Kong,^16^ neither the originators nor the biosimilars of bDMARDs were reimbursed for patients with inadequate methotrexate response (unless patients had fulfilled the local financial assistance program’s clinical and income eligibility requirements). Consequently, most patients were required to pay out of pocket, which led to considerable drug underuse. CT-P13 (biosimilar infliximab) and ABP-501 (biosimilar adalimumab) are 2 biosimilars approved in Hong Kong since 2020, with a price reduction range from 54% to 87% compared with their reference products. The substantial price reduction is expected to reshape the landscape of biosimilar use and indicate an opportunity to access bDMARDs directly after methotrexate failure. Therefore, we proposed this study to examine the cost-effectiveness of treatment sequences initiated with biosimilars infliximab or adalimumab vs frequently used leflunomide among patients with inadequate methotrexate response to inform biosimilar formulary enlisting decisions.

## Methods

This economic evaluation was granted approval by the institutional review board of the University of Hong Kong/Hospital Authority Hong Kong West Cluster. Patient identification was all anonymized, so patient consent was not required. Methods and results were reported according to the Consolidated Health Economic Evaluation Reporting Standards (CHEERS) reporting guideline.^17^

### Model Overview

We developed a multistate Markov transition model with a model structure referenced from Park et al.^18^ The model simulates the lifetime disease progression of 10 000 hypothetical patients with RA with inadequate methotrexate response from the perspective of the Hong Kong public health care institution. The treatment sequence of RA was adapted under the latest clinical guideline.^4^ Patients were initiated with leflunomide or biosimilar DMARDs as the first-line treatment after failure of methotrexate monotherapy, followed by tumor necrosis factor inhibitors (TNFi) DMARDs, non-TNFi DMARDs, Janus kinase inhibitor (JAKi), and supportive care in sequence (eFigure 1 in Supplement 1). All treatments were assumed to be administered in combination with methotrexate. Pharmacological components of bDMARDs, JAKi, and supportive care were extracted from the latest Hong Kong drug formulary.^16^ Treatment efficacies and costs of TNFi, non-TNFi, JAKi, and supportive care were weighted based on local market share (eTable 1 in Supplement 1).

Patients with inadequate methotrexate response entering the model would either remain on the initial treatment or switch to next-line treatment based on the American College of Rheumatology (ACR) criterion at the end of the first cycle. Afterward, patients either continued to take the treatment or discontinued treatment and progressed through the treatment sequence until entry to the supportive care state (eFigure 1 in Supplement 1). Hypothetical patients were simulated to transit among health states at a cycle length of 6 months. Utilities and costs were discounted at an annual rate of 3.5%. Input parameters for the first-line treatment and subsequent treatments were described in Table 1^7,8,19,20^ and eTable 3 in Supplement 1 separately.

**Table 1.** Model Input Parameters of First-Line Treatment^a^

Description	Base case (SD)	Range	Distribution	Source
**Input parameters of transition probabilities**				
ACR response of the at first cycle, % of patients with ACR scores of 20/50/70				
Biosimilar infliximab	73.4 (7.0)/42.7 (7.8)/20.2 (6.3)	NA	Dirichlet	Yoo et al,^19^ 2013
Biosimilar adalimumab	74.6 (7.0)/49.2 (8.1)/26 (7.1)	NA	Dirichlet	Cohen et al,^20^ 2017
Leflunomide	62.0 (4)/38.0 (4)/7.0 (2.1)	NA	Dirichlet	Fleischmann et al,^7^ 2014
Discontinuation probability other than first cycle of biosimilar or leflunomide, %				
Biosimilar infliximab	15.4 (2.5)	12.8-18.4	β	CDARS
Biosimilar adalimumab	12.9 (2.2)	10.7-15.4	β	CDARS
Leflunomide	36.5 (9.1)	19.8-55.2	β	Geborek et al,^8^ 2002
Probability of pneumonia, %				
Biosimilar infliximab	0.74 (0.56)	0.39-1.39	β	CDARS
Biosimilar adalimumab	0.38 (0.33)	0.20-0.76	β	CDARS
Probability of herpes zoster, %				
Biosimilar infliximab	0.16 (0.45)	0.05-0.58	β	CDARS
Biosimilar adalimumab	0.19 (0.27)	0.08-0.49	β	CDARS
Probability of tuberculosis, %				
Biosimilar infliximab	0.16 (0.45)	0.05-0.58	β	CDARS
Biosimilar adalimumab	0.24 (0.27)	0.11-0.56	β	CDARS
Probability of hepatitis B, %				
Biosimilar infliximab	0	NA	β	CDARS
Biosimilar adalimumab	0.14 (0.27)	0.05-0.0042	β	CDARS
**Input parameters of HAQ-DI**				
Baseline HAQ-DI	1.6 (NA)	1.2-2.0	±25% of Mean	Yoo et al,^19^ 2013
HAQ-DI changes in the first cycle of initiation of biosimilar or leflunomide				
<ACR20^b^	−0.16 (0.04)	−0.25 to −0.09	γ	Lee et al,^21^ 2015
ACR20-50^b^	−0.45 (0.1125)	−0.70 to −0.26	γ	Lee et al,^21^ 2015
ACR50-70^b^	−0.70 (0.175)	−1.08 to −0.40	γ	Lee et al,^21^ 2015
≥ACR70^b^	−1.02 (0.255)	−1.58 to −0.58	γ	Lee et al,^21^ 2015
**Input parameters of cost in 2022, US$**				
Drug acquisition cost (per cycle)	NA	NA	NA	NA
Biosimilar infliximab (3 mg/kg at weeks 0, 2, 6, and each 8 weeks)^b^				
First cycle^c^	2792 (698)	1595-4317	γ	HA
Subsequent cycle	1654 (414)	945-2557	γ	HA
Biosimilar adalimumab (40 mg every other week subcutaneous)^b^	940 (235)	537-1453	γ	HA
Leflunomide daily, 20 mg^b^	124 (31)	70-191	γ	HA
Adverse event–related costs (per episode)				
Pneumonia^b^	4983 (1246)	2848-7705	γ	CDARS
Herpes zoster^b^	4546 (1137)	2598-7029	γ	CDARS
Tuberculosis^b^	7043 (1761)	4025-10 890	γ	CDARS
Hepatitis B^b^	2471 (618)	1412-3820	γ	CDARS

Abbreviations: ACR, American College of Rheumatology; CDARS, Clinical Data Analysis and Reporting System; HA, Hospital Authority (Hong Kong); HAQ-DI, Health Assessment Questionnaire Disability Index; NA, not applicable.

^a^ All treatments were used concomitantly with 15 mg methotrexate once weekly.

^b^ SDs were assumed to be 25% of mean.

^c^ Treatment with loading dose different from maintenance dose; the cost of first cycle and subsequent cycles were different.

The model was constructed in R version 4.1.1 (R Project for Statistical Computing) with package *heemod*.^22^ Model construction and input parameter generation were cross-checked independently by 3 coauthors (K.P., Y.W., and Y.S.J.) for quality control.

### Patient Profile and Transition Probability

#### Local Electronic Medical Records Database

A territory-wide electronic medical records (EMR) database, the Clinical Data Analysis and Reporting System (CDARS), was adapted to generate input parameters from the real-world setting wherever suitable. CDARS was operated by the Hospital Authority, a statutory body that manages all public hospitals and clinics and is publicly accessible to all Hong Kong residents (7 million).^23^ A retrospective cohort of patients diagnosed with RA between 2000 and 2021 was retrieved from CDARS using the *International Classification of Diseases, Ninth Revision, Clinical Modification *code (714.0). The RA cohort identified from CDARS was used to determine the patient’s baseline characteristics and market share of included treatments in 2022 (eTable 1 in Supplement 1).

#### Transitional Probability

We applied the ACR20 response rates as the clinical treatment target. Patients continued to take first-line treatment if they achieved the ACR20 criterion or above (ACR50/ACR70). Otherwise, they were switched to the next-line treatment. The ACR20 response rates of the first-line treatment were sourced from the landmark clinical trial.^7,19,20^ Discontinuation rate at the cycles other than the first cycle of first-line treatment and probability of adverse event occurrence during treatment were generated from the CDARS, with computation details provided in eTable 2 in Supplement 1. Baseline mortality rate was extracted from the Hong Kong life table.^24^ It was further multiplied by the risk adjustment factor, which is positively associated with the Health Assessment Questionnaire–Disability Index (HAQ-DI) scores (adjusted mortality = general mortality × 1.33^HAQ-DI^).^25,26^ HAQ-DI is the most widely used measure of function and disability in patients with RA; the score ranges from 0 to 3, with a greater score indicating a worse disease condition.^27^

### Utility

Utilities for health states across disease activity were measured in quality-adjusted life-years (QALYs). We used the HAQ-DI as a proxy to generate the corresponding QALYs at each health state with the formula: QALY = 0.74 − 0.17 × HAQ-DI.^28^ HAQ-DI change was determined by the ACR criterion using individual level data derived from a published randomized clinical trial (RCT)^21,29^ for the first cycle of the first-line treatment. HAQ-DI improvements in other cycles were sourced from the efficacy at 6 months from the corresponding RCTs.^29,30,31,32,33,34,35,36,37,38,39,40,41,42,43^ HAQ-DI changes for TNFi treatment were classified into TNFi experienced and naïve group. HAQ-DI scores were assumed to be unchanged during treatment, given that no significant changes in the score were identified beyond the initial 6 months from the majority of RCTs.^44^

Disease condition was expected to deteriorate continuously for patients receiving supportive care. The HAQ-DI score changes were −0.04 and 0.2 in the first and second cycles, followed by 0.28 at every other cycle until it reached the upper bound of the HAQ-DI score.^44^ We assumed that patients’ HAQ-DI improvements would disappear upon treatment discontinuation, and the HAQ-DI score would bounce back to the beginning of the treatment.

### Costs

#### Drug Acquisition Costs

Drug unit costs were extracted from the Hospital Authority Pharmacy Management System. We calculated the drug acquisition costs per treatment course based on recommended dosage and adjusted these to fit the cycle length of the Markov model.

#### Adverse Event–Related Costs

Four severe adverse events (pneumonia, herpes zoster, tuberculosis, hepatitis B) associated with bDMARDs or JAKi treatments resulting in hospitalization and substantial health care costs were considered in the model (eTable 4 in Supplement 1).

#### Supportive Care Nonpharmacological Costs

The cohort with supportive care was expected to receive additional treatment with intraarticular steroid injections every 6 months (up to twice in a lifetime) and visit a community-allied physiotherapy health service biweekly to mitigate the symptoms.^45^ All costs are presented in US dollars in 2022 (exchange rate was US $1 = HK $7.78 in 2022^46^).

### Statistical Analysis

#### Base-Case Analysis

Incremental costs, incremental QALY, and incremental cost-effectiveness ratio (ICER) were computed by comparing treatment sequences initiated with biosimilar DMARDs vs leflunomide. ICERs were subsequently compared against a willingness-to-pay threshold (WTP) of a 1-time gross domestic product (GDP) per capita in Hong Kong in 2022^47^ (US $48 555 per QALY gain), according to the World Health Organization recommendation.^48^ Treatment sequences with ICERs below the WTP threshold were considered cost-effective.

#### Sensitivity Analyses

Deterministic sensitivity analysis was conducted by varying each parameter according to its 95% CI independently. In probabilistic sensitivity analysis, all tested parameters were varied simultaneously with a predefined distribution for 10 000 iterations. For parameters where SDs were not available, we applied a 25% mean value as the SD for simulation. The cost-effective acceptability curve (CEAC) displayed the probability of each comparator being a cost-effective strategy within a range of WTP threshold (US $0-$80 000).

#### Scenario Analysis

We conducted several scenario analyses to capture the effect of parameter uncertainty and modeling structure on cost-effective conclusions. First, various mapping algorithms converting HAQ-DI score to utility were applied to eliminate bias from the algorithm selection.^49^ Second, we assessed the ICER variation regarding different model durations (5-40 years), discounting rates (0%-5%), and population baseline age (56 ± 5 years). In addition, a simplified treatment sequence where patients were initiated with receiving leflunomide or biosimilar DMARDs were followed by supportive care directly to reflect clinical practice, considering that no bDMARDs or JAKi were reimbursed by the public health care payer in Hong Kong.^16^ Last, in settings where the public health care payer does not cover physiotherapies, we removed nonpharmacological costs of supportive care.

#### Supplementary Cost Analysis From a Global Context

The unit cost of health care intervention was the most frequently cited cause of variation in economic evaluation across different contexts.^50^ Considering the marginal cost of leflunomide, the cost-effectiveness of the treatment sequence initiated with biosimilar DMARDs was largely driven by the price of biosimilars. In this supplement analysis, we used global infliximab sales data in July 2022 from the IQVIA Multinational Integrated Data Analysis System (IQVIA-MIDAS) database to depict the distribution of biosimilar infliximab prices globally. The database has been widely used in analyzing global pharmaceutical sales and costs trend.^51,52^ We select infliximab and its biosimilars given their considerable global sales and broader market availability.^53^ Countries with at least 1 biosimilar infliximab on sale in July 2022 were included in the analysis. Countries were further stratified by income level according to World Bank income classifications.^54^ Statistical analysis was performed using R version 4.1.1 (R Project for Statistical Computing) from January 2023 to March 2024

## Results

### Base-Case Analysis

In total, 25 099 patients with RA were identified (mean [SD] age: 56 [17] years; 19 469 women [72.7%] and 5630 men [27.3%]) (eTable 1 in Supplement 1)]. For leflunomide, the lifetime health care costs and QALYs for were US $154 632 and 14.82 QALYs, respectively; for biosimilar infliximab, they were US $152 326 and 15.35 QALYs, respectively, and for biosimilar adalimumab, they were US $145 419 and 15.55 QALYs, respectively (Table 2). Both treatment sequences initiated with biosimilar DMARDs demonstrated lower costs and greater QALYs compared with treatment sequence initiated with leflunomide. Biosimilar infliximab was associated with greater health care costs (US $6907) but a lower QALY gain (−0.20) compared with biosimilar adalimumab.

**Table 2.** Base-Case Results

Strategy	Cost, US$^a^		QALY		ICER (US$/QALY)^a^
	Net	Incremental	Net	Incremental	
Leflunomide	154 632	Reference	14.82	Reference	Reference
Biosimilar infliximab	152 326	−2306	15.35	0.53	−4351
Biosimilar adalimumab	145 419	−9213	15.55	0.73	−12 621

Abbreviations: ICER, Incremental cost-effectiveness ratio; QALY, quality-adjusted life-year.

^a^ The exchange rate was US $1 = HK $7.78 in 2022.

### Sensitivity Analyses

**Figure 1.**
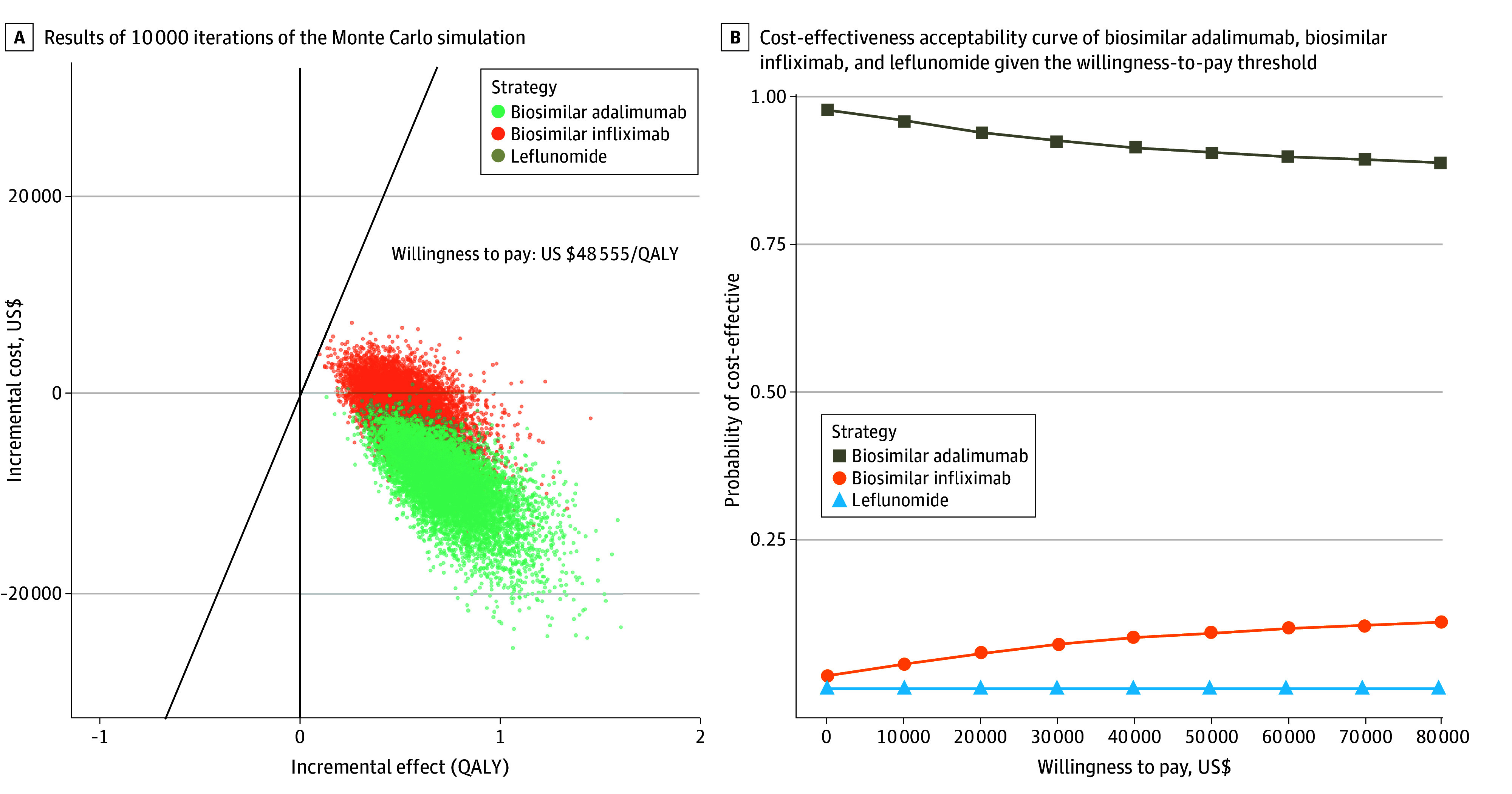
Probability Sensitivity Analysis A, Results of 10 000 iterations of the Monte Carlo simulation for biosimilar adalimumab (ABP-501), biosimilar infliximab (CT-P13), and leflunomide. B, Cost-effectiveness acceptability curve of biosimilar adalimumab, biosimilar infliximab, and leflunomide given the willingness to pay threshold of US $48 555/QALY gain. QALY indicates quality-adjusted life-year.

The top 10 parameters identified from the deterministic sensitivity analysis with the greatest effect on ICER ranges were presented in tornado diagrams in descending order (eFigures 2, 3, and 4 in Supplement 1). The ICER range (USD/QALY) was −$9088 to $10 238 for comparison pair biosimilar infliximab vs leflunomide; −$15 797 to −$8615 for biosimilar adalimumab vs leflunomide; and −$108 903 to −$21 333 for biosimilar adalimumab vs biosimilar infliximab. Varying all parameters independently over a predefined range, the corresponding ICERs were consistently lower than the WTP threshold of US $48 555/QALY gain. The cost-effectiveness conclusion remains unchanged in the deterministic sensitivity analysis. In the probabilistic sensitivity analysis, the probability of treatment sequences initiated with leflunomide, biosimilar infliximab, and biosimilar adalimumab being a cost-effective strategy out of 10 000 iterations was 0%, 9%, and 91% at the predefined WTP threshold (Figure 1).

### Scenario Analysis

**Figure 2.**
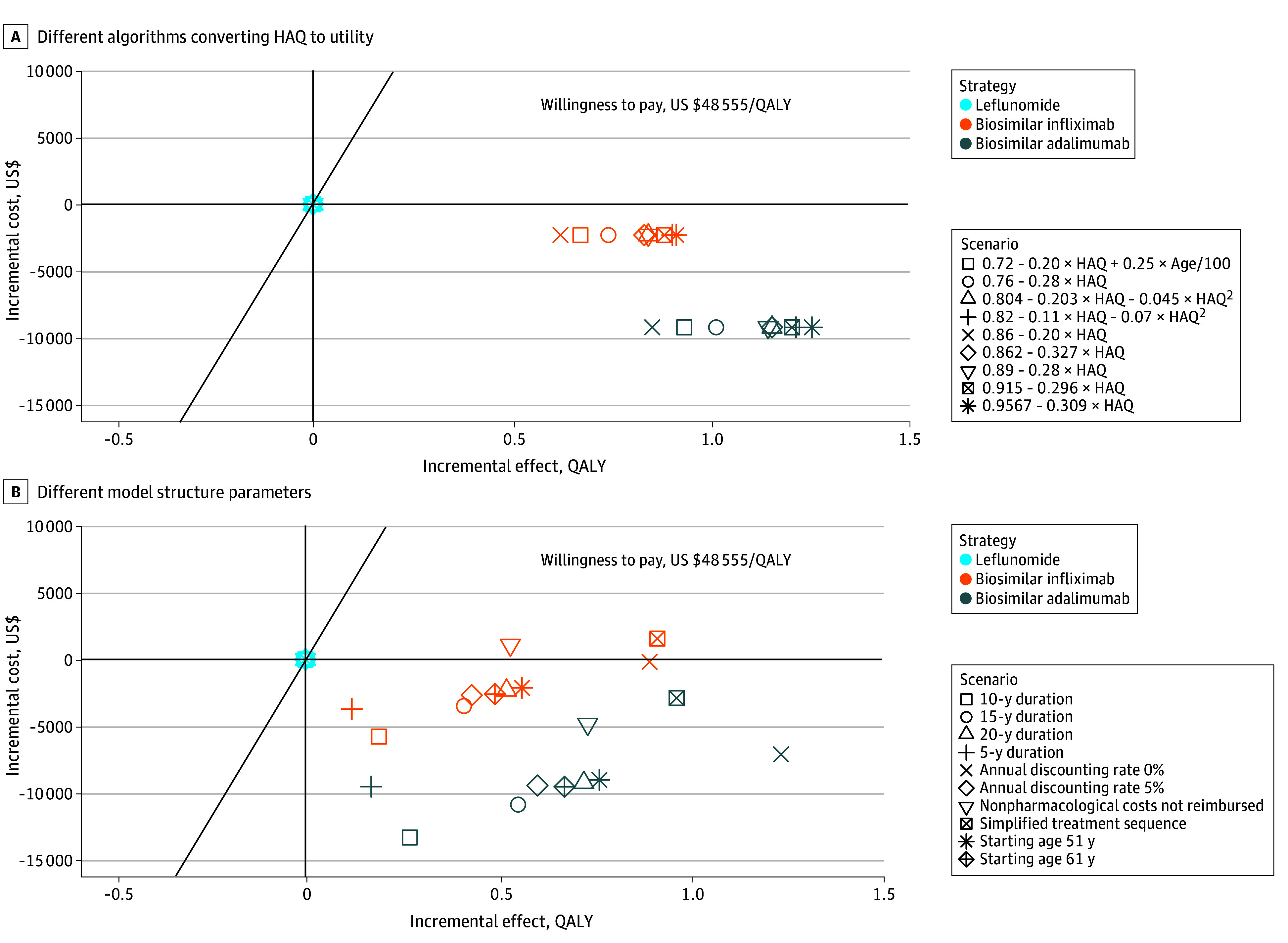
Scenario Analysis Varying Converting Formula and Model Structure HAQ indicates Health Assessment Questionnaire; QALY, quality-adjusted life-year.

Both biosimilar infliximab and biosimilar adalimumab remained a cost-effective alternative to leflunomide when changing the mapping algorithm of HAQ-DI, model simulation time, cohort starting age, discounting rate, treatment sequence, or ignoring nonpharmacological costs of supportive care (Figure 2). The numerical results of scenario analysis were presented in eTable 5 in Supplement 1.

#### Cost of Biosimilar From a Global Context

**Figure 3.**
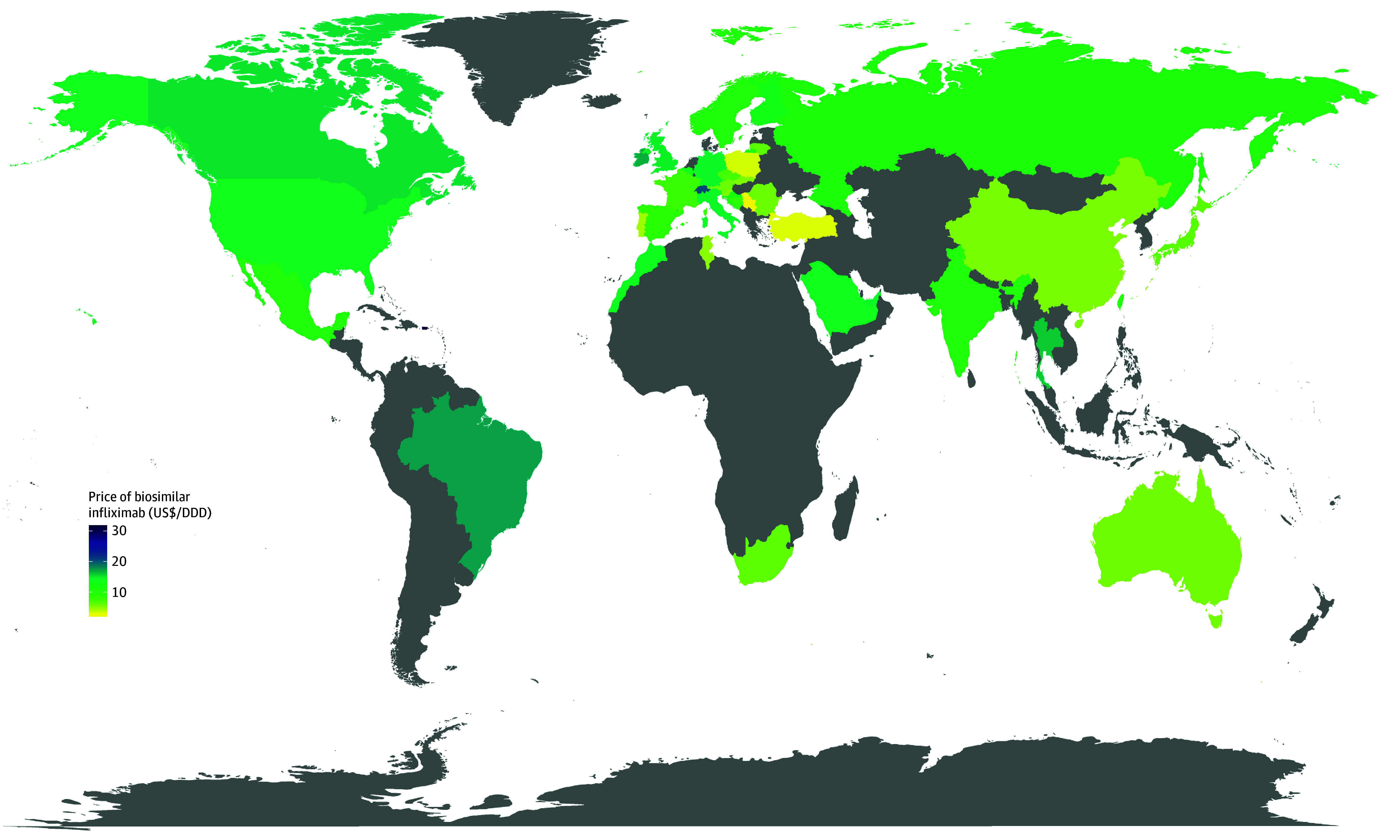
Global Price of Biosimilar Infliximab in July 2022 DDD indicates daily defined dose. Data in gray areas are not available in the IQVIA-MIDAS database.

We identified 41 countries with recorded sales data of biosimilar infliximab in July 2022 from the IQVIA-MIDAS database (Figure 3). Biosimilar infliximab was identified in 28 high-income countries (median cost: US $10.46/daily defined dose [DDD]); 10 upper middle-income countries (median cost: US $7.37/DDD); and 3 lower middle-income countries (median cost: US $10.51/DDD). We identified 15 out of the 28 high-income countries that have a similar level of economic development as Hong Kong and lower unit price of biosimilar infliximab (eTable 6 in Supplement 1).

## Discussion

To our knowledge, this is the first cost-effective analysis evaluating the treatment sequence initiated with biosimilar DMARDs after inadequate methotrexate response among patients with RA. Park et al^18^ found it is cost-effective to apply treatment sequence initiated with etanercept originator vs leflunomide with an ICER US $8050/QALY gain in Korea in 2016.^18^ While in the base-case result, we found that the treatment sequence initiated with biosimilar DMARDs was associated with improved QALY and reduced health care costs, leading to a negative ICER value. Two reasons could explain such disparity. First, the price difference between biosimilars and bio-originator was dramatic in Hong Kong (biosimilar adalimumab: US $940 vs adalimumab originator: US $7073 [87% reduction]; biosimilar infliximab: US $1654 vs infliximab originator: US $3609 [54% reduction]). Second, the cost of supportive care (US $2891 per course) in Hong Kong is much higher than the medication cost. Treatment sequence initiated with biosimilar DMARDs slow down disease progression, therefore reducing the time patients spend on supportive care and eventually lead to lower overall treatment costs.

Biological DMARDs underuse has been widely reported worldwide due to stringent reimbursement criteria, such as minimum disease duration and activity,^55^ where countries with high socioeconomic welfare tend to have more flexible criteria.^56^ Hong Kong is a highly developed economic entity ranked 19th among the major economies,^57^ yet its reimbursement for bDMARDs lags behind other Asian areas with comparable universal health coverage and economic levels such as Japan,^58^ Taiwan,^59^ and Korea,^60^ where at least 1 bDMARD is available after the failure of methotrexate. In the scenario analysis, the simplified treatment sequence resulted in rapid transition to supportive care state, ended up with substantial reduction in QALYs (biosimilar adalimumab decreased from 15.55 to 9.66; biosimilar infliximab decreased from 15.35 to 9.61; leflunomide decreased from 14.82 to 8.70). Although the incremental QALYs comparing biosimilar DMARDs vs leflunomide remain considerable (biosimilar adalimumab: 0.96, biosimilar infliximab: 0.91), Our findings suggest that under the premise of an acceptable budget and well-evaluated opportunity costs, Hong Kong’s local health care agencies should actively promote the inclusion of biosimilar DMARDs in the medical insurance catalog.

Our study has several merits. First, different from the clinical practice setting, RCT participants generally have better treatment compliance and limited follow-up time.^61^ We overcame the aforementioned deficiencies by generating input parameters from local electronic medical records wherever appropriate to reflect the real-world scenarios after the deployment of biosimilar DMARDs. Second, the current model incorporated treatment sequence by mode of action, instead of single drug alone, which is consistent with the clinical guideline recommendation. It reflects realistic clinical practice where patients can opt into receiving different drugs and thus improved the model’s generalizability to broader context. Third, we classified patients into TNFi experienced and naive groups to incorporate the influence of previous treatment exposure, which rendered a more accurate estimation of the efficacies of subsequent treatments.

### Limitations

This study has limitations. In the absence of local evidence, treatment efficacies were mainly retrieved from independent RCTs with heterogeneity in patients’ demographic and clinical profiles. Also, we used results from landmark trials to generate the HAQ-DI improvements for the second- (TNFi), third- (non-TNFi), and fourth- (JAKi) line treatments in the model. However, in trial settings, the study population was mainly naive to bDMARDs or experienced only 1 bDMARD before recruiting. Treatment efficacy from trials was expected to be more prominent than our simulated patient cohort because the latter would experience multiple treatment failures with worse disease conditions.^62^ Therefore, applying HAQ-DI improvements from RCTs to the simulated cohort might overestimate the treatment efficacy. Nevertheless, the sensitivity and scenario analysis have indicated that the potential variation in these data did not affect the cost-effectiveness conclusion. Additionally, chronic inflammatory arthritis-induced disability is linked to loss of work capacity and early retirement.^63^ Prescribing patients with effective bDMARDs can improve patients’ work capacity^64^ for its higher remission rates and long-term quality of life compared with csDMARDs. Considering the monetary savings from retained employment and work capacity, we anticipate that our model underestimated the cost benefits of treatment sequence initiated with biosimilar DMARDs based on published cost-effectiveness studies that incorporated productivity loss.^65,66^

Although our study conclusion cannot be directly applied in other health care settings beyond Hong Kong due to the common drawback of system-specific health economic studies, the deterministic sensitivity analysis (eFigures 2 and 3 in Supplement 1) has indicated that the cost of biosimilar DMARDs was the primary driver of cost-effective conclusion. The supplement analysis from the global context showed that the unit cost of biosimilar infliximab in Hong Kong was higher than the median cost among high-income countries. Countries with a similar WTP threshold (assuming 1-unit GDP per capita as the WTP threshold) as Hong Kong,^48^ such as Australia, Norway, and Sweden, would likely benefit from using biosimilar DMARDs as the local market provides a lower price. The cost-effectiveness of treatment sequence initiated with biosimilar DMARDs in these jurisdictions is probable and worth further investigation.

## Conclusions

From the Hong Kong Public institution perspective, treatment sequences initiated with biosimilar DMARDs were cost-effective compared with treatment sequence initiated with leflunomide among patients with RA and an inadequate methotrexate response. This study can serve to inform health care stakeholders, rheumatologists, and patients with the unmet needs of bDMARDs about the benefits and financial feasibility of biosimilar DMARDs.

## Notes

Supplement 1. **eTable 1.** Patients’ Demographics and Disease Modifying Antirheumatic Drugs Market Share in Hong Kong **eTable 2.** Number of Patients Discontinue Treatment or Experience Adverse Events During Two Years Follow-Up **eTable 3.** Model Input Parameters of Subsequent Treatments **eTable 4.** Cost of Adverse Events per Episode **eTable 5.** Numerical Results of Scenario Analysis **eTable 6.** Price of Biosimilar Infliximab in July 2022 **eFigure 1.** Treatment Transition Pathway in the Markov Simulation Model **eFigure 2.** Deterministic Sensitivity Analysis Comparing CT-P13 Against LEF **eFigure 3.** Deterministic Sensitivity Analysis Comparing ABP-501 Against LEF **eFigure 4.** Deterministic Sensitivity Analysis Comparing ABP-501 Against CT-P13

Supplement 2.
Data Sharing Statement
