# Supplementary material for: Cost-Effectiveness of Biosimilars vs Leflunomide in Patients With Rheumatoid Arthritis
Source: JAMA Netw Open. 2024 Jun 26;7(6):e2418800. doi: 10.1001/jamanetworkopen.2024.18800 (PMC11208978; doi:10.1001/jamanetworkopen.2024.18800)
Supplement: Supplement 1. — eTable 1. Patients’ Demographics and Disease Modifying Antirheumatic Drugs Market Share in Hong Kong eTable 2. Number of Patients Discontinue Treatment or Experience Adverse Events During Two Years Follow-Up eTable 3. Model Input Parameters of Subsequent Treatments eTable 4. Cost of Adverse Events per Episode eTable 5. Numerical Results of Scenario Analysis eTable 6. Price of Biosimilar Infliximab in July 2022 eFigure 1. Treatment Transition Pathway in the Markov Simulation Model eFigure 2. Deterministic Sensitivity Analysis Comparing CT-P13 Against LEF eFigure 3. Deterministic Sensitivity Analysis Comparing ABP-501 Against LEF eFigure 4. Deterministic Sensitivity Analysis Comparing ABP-501 Against CT-P13 [file jamanetwopen-e2418800-s001.pdf]

Supplemental Online Content

Peng K, Chan SW, Wang Y, et al. Cost-effectiveness of biosimilars vs leflunomide in patients with rheumatoid arthritis. *JAMA Netw Open.* 2024;7(6):e2418800. doi:10.1001/jamanetworkopen.2024.18800

eTable 1. Patients' Demographics and Disease Modifying Antirheumatic Drugs Market Share in Hong Kong

eTable 2. Number of Patients Discontinue Treatment or Experience Adverse Events During Two Years Follow Up

eTable 3. Model Input Parameters of Subsequent Treatments

eTable 4. Cost of Adverse Events Per Episode

eTable 5. Numerical Results of Scenario Analysis

eTable 6. Price of Biosimilar Infliximab in July 2022

eFigure 1. Treatment Transition Pathway in the Markov Simulation Model

eFigure 2. Deterministic Sensitivity Analysis Comparing CT-P13 Against LEF

eFigure 3. Deterministic Sensitivity Analysis Comparing ABP-501 Against LEF

eFigure 4. Deterministic Sensitivity Analysis Comparing ABP-501 Against CT-P13

eReferences

This supplemental material has been provided by the authors to give readers additional information about their work.

© 2024 Peng K et al. *JAMA Network Open.*

eTable 1: Patients' demographics and disease modifying antirheumatic drugs market share in Hong Kong

Description	Value
**Number of included patients**	25,099
**Demographic**	
Age (Years)	56
Women (%)	72.7
Body weight (kg)	58
**Market share (%)**	
TNFi	
Adalimumab	18.8
Certolizumab Pegol	14.5
Etanercept	36.2
Golimumab	29.2
Infliximab	1.4
Non-TNFi	
Abatacept	16.1
Rituximab	14.9
Sarilumab	11.1
Tocilizumab	58.0
JAKi	
Tofacitinib	71.1
Baricitinib	28.9
Supportive treatment	
Methotrexate	56.7

© 2024 Peng K et al. *JAMA Network Open.*

Leflunomide	15.1
Cyclosporine	2
Hydroxychloroquine	25.5
Azathioprine	0.7

TNFi: Tumour Necrosis Factor inhibitors; JAKi: Janus kinase inhibitor.

© 2024 Peng K et al. *JAMA Network Open.*

eTable 2: Number of patients discontinue treatment or experience adverse events during two years follow up

Description	Number of patient initiated with treatment	Number of patient discontinue the treatment/experience adverse events after two years
**Discontinuation**		
CT-P13	209	107
ABP-501	240	138
TNFi	1586	985
Non-TNFi	905	607
JAKi	471	327
**Pneumonia**		
CT-P13	309	9
ABP-501	523	8
TNFi	2324	42
Non-TNFi	1439	40
JAKi	864	14
**Herpes zoster**		
CT-P13	309	2
ABP-501	523	4
TNFi	2324	19
Non-TNFi	1439	27
JAKi	864	19
**Tuberculosis**		
CT-P13	309	2
ABP-501	523	5

© 2024 Peng K et al. *JAMA Network Open.*

TNFi	2324	10
Non-TNFi	1439	5
JAKi	864	1
**Hepatitis B**		
CT-P13	309	0
ABP-501	523	3
TNFi	2324	12
Non-TNFi	1439	20
JAKi	864	10

TNFi: Tumour Necrosis Factor Inhibitors; JAKi: Janus kinase inhibitor.

Probability of treatment discontinuation and adverse events were derived by analysing the prescription and diagnosis records of the rheumatoid arthritis cohort. We divided the number of patients who discontinued treatment/experienced adverse events during the follow-up period with the number of patients initiated on the corresponding treatment. The cumulative probability P1 was further converted to the six-month probability to fit the model structure using the following formula: P2 = 1-(1-P1) ^ (1/(t1/t2)), where P1, t1, and P2, t2 are probability and time duration corresponding to observation duration (two years) and cycle duration (six months), assuming that the probability followed an exponential distribution.

© 2024 Peng K et al. *JAMA Network Open.*

eTable 3: Model input parameters of subsequent treatments

Description	Base case	Standard deviation	Lower bound	Upper bound	Distribution	Source
**Input parameters of transition probabilities**						
**Discontinuation probability other than first cycle of biosimilar or LEF (%)**						
**TNFi**	11.2	0.8	10.4	12.1	Beta	CDARS
**Non-TNFi**	9.5	1	8.5	10.6	Beta	CDARS
**JAKi**	8.7	1.3	7.4	10.2	Beta	CDARS
**Adverse events probability (%)**						
Pneumonia						
**TNFi**	0.45	0.15	0.34	0.61	Beta	CDARS
**Non-TNFi**	0.7	0.23	0.52	0.95	Beta	CDARS
**JAKi**	0.41	0.26	0.24	0.68	Beta	CDARS
Herpes Zoster						
**TNFi**	0.21	0.11	0.13	0.32	Beta	CDARS
**Non-TNFi**	0.47	0.20	0.33	0.69	Beta	CDARS
**JAKi**	0.55	0.28	0.36	0.86	Beta	CDARS
Tuberculosis						
**TNFi**	0.11	0.09	0.06	0.20	Beta	CDARS
**Non-TNFi**	0.09	0.10	0.04	0.20	Beta	CDARS
**JAKi**	0.03	0.12	0.01	0.16	Beta	CDARS
Hepatitis B						
**TNFi**	0.13	0.09	0.07	0.0023	Beta	CDARS

© 2024 Peng K et al. *JAMA Network Open.*

**Non-TNFi**	0.35	0.17	0.23	0.0054	Beta	CDARS
**JAKi**	0.29	0.23	0.16	0.0053	Beta	CDARS
**Input parameters of HAQ-DI**						
**HAQ-DI changes for Supportive care**						
**first cycle***	-0.04	0.01	-0.06	-0.02	Gamma	[1]
**second cycle***	0.20	0.05	0.11	0.31	Gamma	[1]
**subsequent cycle***	0.28	0.07	0.16	0.43	Gamma	[1]
**HAQ-DI changes for TNFi among TNFi naïve patients*, ****	-0.57	0.14	-0.88	-0.33	Gamma	
**Adalimumab**	-0.56					[2]
**Infliximab**	-0.51					[3]
**Golimumab**	-0.38					[4]
**Etanercept**	-0.7					[5]
**Certolizumab Pegol**	-0.63					[6]
**HAQ-DI changes for TNFi among TNFi experienced patients*, ****	-0.35	0.09	-0.54	-0.20	Gamma	
**Adalimumab**	-0.51					[7]
**Infliximab**	-0.22					[8]
**Golimumab**	-0.30					[9]
**Etanercept**	-0.31					[10]
**Certolizumab Pegol**	-0.37					[11]
**HAQ-DI changes for non-TNFi among TNFi experienced patients*, ****	-0.43	0.102	-0.66	-0.25	Gamma	
**Abatacept**	-0.51					[12]

© 2024 Peng K et al. *JAMA Network Open.*

**Rituximab**	-0.4					[13]
**Sarilumab**	-0.58					[14]
**Tocilizumab**	-0.39					[15]
**HAQ-DI changes for JAKi among TNFi experienced patients*, ****	-0.41	0.102	-0.63	-0.23	Gamma	
**Tofacitinib**	-0.43					[16]
**Baricitinib**	-0.36					[17]
**Input parameters of cost (2022 USD)**						
**TNFi *, ****						
**First cycle*****	5065	1266	2895	7831	Gamma	HA
**Subsequent cycle**	5030	1258	2875	7777	Gamma	HA
**Adalimumab (40mg every other week)**	7073					HA
**Infliximab (3mg/kg at weeks 0, 2, 6, and each 8 weeks)**						
**First cycle*****	6100					HA
**Subsequent cycle**	3609					HA
**Golimumab (50mg every four weeks)**	6210					HA
**Etanercept (25mg per week subcutaneous)**	3144					HA
**Certolizumab Pegol (200mg every other week)**	4815					HA
**Non-TNFi *, ****						
**First cycle*****	8525	2131	4872	13181	Gamma	HA
**Subsequent cycle**	8193	2048	4683	12668	Gamma	HA
**Abatacept (500mg 3 times at first 4 weeks**						

© 2024 Peng K et al. *JAMA Network Open.*

**and thereafter every 4 weeks)**						
**First cycle*** **	8790					HA
**Subsequent cycle**	6724					HA
**Rituximab (1000mg every 12 week)**	10112					HA
**Sarilumab (200mg every 2 weeks)**	5358					HA
**Tocilizumab (8mg/kg every 4 week)**	8636					HA
**JAKi*, ** **	4433	1108	2533	6854	Gamma	HA
**Tofacitinib (5mg twice a day)**	4518					HA
**Baricitinib (2mg daily)**	4233					HA
**Supportive care – pharmacologic*, ** **	47	12	26	72	Gamma	HA
**MTX (15mg once weekly)**	11					HA
**LEF (20mg daily)**	113					HA
**Cyclosporine (5mg/kg daily)**	523					HA
**Hydroxychloroquine (400mg daily)**	20					HA
**Azathioprine (2mg/kg daily)**	38					HA
**Prednisolone (40mg daily)**	14					HA
**Celecoxib (400mg daily)**	52					HA
**Supportive care non-pharmacological ***						
**First three cycles**	3547	887	2027	5484	Gamma	HA
**Subsequent cycle**	2891	723	1652	4470	Gamma	HA
**Intra-articular steroid injections (Up to two times)**	656					
**Physiotherapy clinics visit (Bi-weekly)**	2891					

ACR: American College of Rheumatology; CDARS: Clinical Data Analysis and Reporting System. LEF: Leflunomide; TNFi: Tumour Necrosis Factor Inhibitors; JAKi: Janus kinase inhibitor; HAQ-DI:

© 2024 Peng K et al. *JAMA Network Open.*

Health Assessment Questionnaire Disability Index. All treatments were used concomitantly with 15mg methotrexate once weekly.

*Standard deviations were assumed to be 25% of mean.

**Integrated values of TNFi/non-TNFi/JAKi/supportive pharmacology were calculated based on market share presented in eTable 1.

***Treatment with loading dose different from maintenance dose, the cost of first cycle and subsequent cycles were different.

© 2024 Peng K et al. *JAMA Network Open.*

eTable 4: Cost of adverse events per episode

	Herpes Zoster	Pneumonia	Tuberculosis	Hepatitis B
Mean length of hospitalisation - General (Days)	6.17	6.44	10.12	3.56
Mean length of hospitalisation - Intensive care (Days)	0.16	0.21	0.08	0.04
Mean length of hospitalisation - High dependency (Days)	0	0.06	0.09	0
Hospitalisation costs (USD)	4546	4983	7043	2471

Daily costs of general ward, intensive care ward, and high dependency ward are USD 657, USD 3144, USD 1759, respectively, sourced from Hong Kong gazette - Fees and Charges[18]. Cost per adverse event episode was calculated by multiplying mean length of hospitalisation analysed using inpatient records with the daily hospitalisation charge.

© 2024 Peng K et al. *JAMA Network Open.*

eTable 5: Numerical results of scenario analysis

Scenario	Strategy	Net cost	Incremental Cost	Net QALY	Incremental QALY	ICER
0.804 – 0.203 × HAQ – 0.045 × HAQ^2	LEF	154,632	Ref	11.47	Ref	
	CT-P13	152,326	-2306	12.31	0.84	-2745
	ABP-501	145,419	-9213	12.62	1.15	-8011
0.82 – 0.11 × HAQ – 0.07 × HAQ^2	LEF	154,632	Ref	13.72	Ref	
	CT-P13	152,326	-2306	14.62	0.9	-2562
	ABP-501	145,419	-9213	14.93	1.21	-7614
0.89 – 0.28 × HAQ	LEF	154,632	Ref	13.49	Ref	
	CT-P13	152,326	-2306	14.33	0.84	-2745
	ABP-501	145,419	-9213	14.63	1.14	-8082
0.76 – 0.28 × HAQ	LEF	154,632	Ref	9.88	Ref	
	CT-P13	152,326	-2306	10.62	0.74	-3116
	ABP-501	145,419	-9213	10.89	1.01	-9122
0.86 – 0.20 × HAQ	LEF	154,632	Ref	17.09	Ref	
	CT-P13	152,326	-2306	17.71	0.62	-3719
	ABP-501	145,419	-9213	17.94	0.85	-10,839
0.915 – 0.296 × HAQ	LEF	154,632	Ref	13.43	Ref	
	CT-P13	152,326	-2306	14.31	0.88	-2620
	ABP-501	145,419	-9213	14.63	1.2	-7677
0.9567 – 0.309 × HAQ	LEF	154,632	Ref	14.05	Ref	
	CT-P13	152,326	-2306	14.96	0.91	-2534

© 2024 Peng K et al. *JAMA Network Open.*

	ABP-501	145,419	-9213	15.3	1.25	-7370
0.862 – 0.327 × HAQ	LEF	154,632	Ref	10.91	Ref	
	CT-P13	152,326	-2306	11.74	0.83	-2778
	ABP-501	145,419	-9213	12.06	1.15	-8011
0.72 – 0.20 × HAQ + 0.25 × Age/100	LEF	154,632	Ref	17.45	Ref	
	CT-P13	152,326	-2306	18.12	0.67	-3442
	ABP-501	145,419	-9213	18.38	0.93	-9906
5 years duration	LEF	41,523	Ref	4.84	Ref	
	CT-P13	37,815	-3708	4.96	0.12	-30,900
	ABP-501	32,002	-9521	5.01	0.17	-56,006
10 years duration	LEF	85,145	Ref	8.61	Ref	
	CT-P13	79,374	-5771	8.8	0.19	-30,374
	ABP-501	71,820	-13,325	8.88	0.27	-49,352
15 years duration	LEF	132,946	Ref	12.94	Ref	
	CT-P13	129,463	-3483	13.35	0.41	-8495
	ABP-501	122,081	-10,865	13.49	0.55	-19,755
20 years duration	LEF	154,530	Ref	14.82	Ref	
	CT-P13	152,218	-2312	15.34	0.52	-4446
	ABP-501	145,307	-9223	15.54	0.72	-12,810
Annual discounting rate 0%	LEF	236,709	Ref	22.1	Ref	
	CT-P13	236,532	-177	22.99	0.89	-199
	ABP-501	229,608	-7101	23.33	1.23	-5773
Annual discounting rate 5%	LEF	132,173	Ref	12.82	Ref	

© 2024 Peng K et al. *JAMA Network Open.*

	CT-P13	129,498	-2675	13.25	0.43	-6221
	ABP-501	122,736	-9437	13.42	0.6	-15,728
Starting age 51	LEF	164,662	Ref	15.67	Ref	
	CT-P13	162,532	-2130	16.23	0.56	-3804
	ABP-501	155,642	-9020	16.43	0.76	-11,868
Starting age 61	LEF	143,127	Ref	13.83	Ref	
	CT-P13	140,533	-2594	14.32	0.49	-5294
	ABP-501	133,593	-9534	14.5	0.67	-14,230
Non-Pharmacological costs of supportive care not reimbursed	LEF	121,873	Ref	14.82	Ref	
	CT-P13	122,950	1077	15.35	0.53	2032
	ABP-501	117,029	-4844	15.55	0.73	-6636
Simplified treatment sequence	LEF	89,016	Ref	8.7	Ref	
	CT-P13	90,574	1558	9.61	0.91	1712
	ABP-501	86,128	-2888	9.66	0.96	-3008

LEF: Leflunomide; HAQ: Health Assessment Questionnaire; QALY: Quality-adjusted life year; ICER: Incremental cost-effectiveness ratio.

© 2024 Peng K et al. *JAMA Network Open.*

eTable 6: Price of biosimilar infliximab in July 2022

Countries	Price of biosimilar infliximab (USD/DDD)	GDP per capital in 2022 (USD)	Income group
Poland	3.35	18,688	High income
Portugal	4.37	24,515	High income
Slovenia	5.13	28,439	High income
Slovakia	5.14	21,257	High income
Australia	5.58	65,100	High income
Austria	5.76	52,085	High income
Romania	5.80	15,787	High income
Lithuania	6.29	25,065	High income
Japan	6.41	33,824	High income
Czech Republic	6.97	27,223	High income
France	7.54	40,886	High income
Spain	8.96	29,675	High income
Taiwan	10.23	34,050	High income
Sweden	10.30	56,374	High income
Norway	10.63	106,177	High income
Hong Kong	10.80	48,555	High income
Croatia	11.66	18,570	High income
Finland	12.77	50,916	High income
Belgium	12.90	49,927	High income
USA	13.24	76,330	High income
Saudi Arabia	13.42	30,448	High income

© 2024 Peng K et al. *JAMA Network Open.*

Germany	14.36	48,718	High income
United Arab Emirates	14.39	53,708	High income
Italy	14.40	34,776	High income
UK	14.71	46,125	High income
Canada	15.01	54,918	High income
Ireland	16.71	103,983	High income
Switzerland	20.51	93,260	High income
Puerto Rico	31.93	35,209	High income
Serbia	2.01	9538	Upper middle income
Turkey	3.16	10,675	Upper middle income
China	5.31	12,720	Upper middle income
Bulgaria	5.63	13,974	Upper middle income
South Africa	6.26	6766	Upper middle income
Bosnia and Herzegovina	8.48	7569	Upper middle income
Mexico	10.43	11,497	Upper middle income
Russia	10.95	15,271	Upper middle income
Thailand	15.64	6910	Upper middle income
Brazil	17.17	8918	Upper middle income
Tunisia	4.88	3747	Lower middle income
India	10.51	2411	Lower middle income
Morocco	13.89	3442	Lower middle income

DDD: Defined daily dose; USA: United States of America; UK: United Kingdom; GDP: Gross domestic product. IQVIA Multinational Integrated Data Analysis System covers 95% of the global prescription drug market, with coverage rates surpass 80% in majority countries. Countries with at least one biosimilar infliximab on sale in July 2022 were included in the analysis. The sales volume is standardized to World Health Organization Defined Daily Dose (WHODDD) to facilitate cross-region comparison (3.75 mg per day for infliximab). The sales data are collected at manufacture price level, trade price level, and national health reimbursement level depending on the purchase channel and market. The sales are expressed in US dollar at the exchange rates in effect at the time the sales were made.

© 2024 Peng K et al. *JAMA Network Open.*

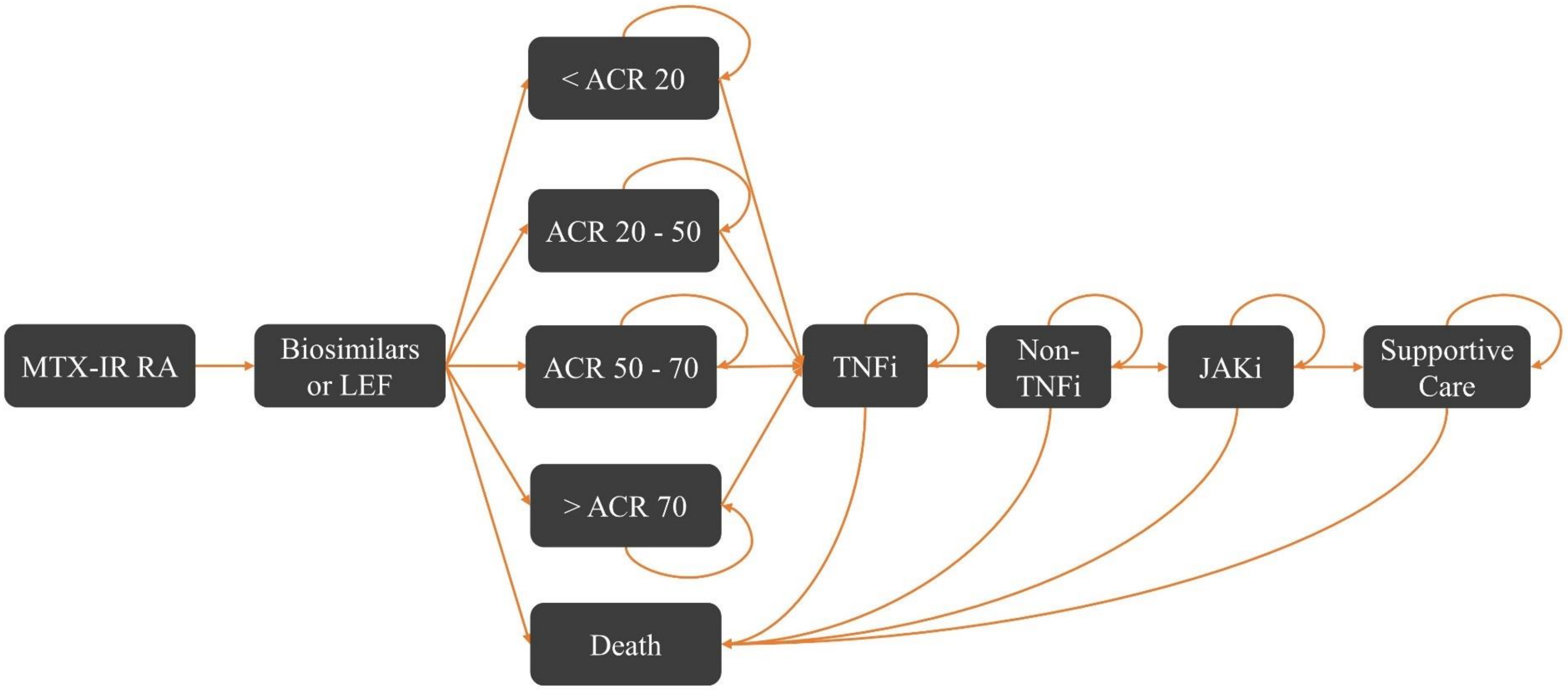

eFigure 1: Treatment transition pathway in the Markov simulation model

MTX-IR RA: Rheumatoid arthritis patients with inadequate response to Methotrexate; Biosimilars include ABP-501 and CT-P13; ACR: American College of Rheumatology; TNFi: Tumour necrosis factor inhibitors; JAKi: Janus kinase inhibitors

© 2024 Peng K et al. *JAMA Network Open.*

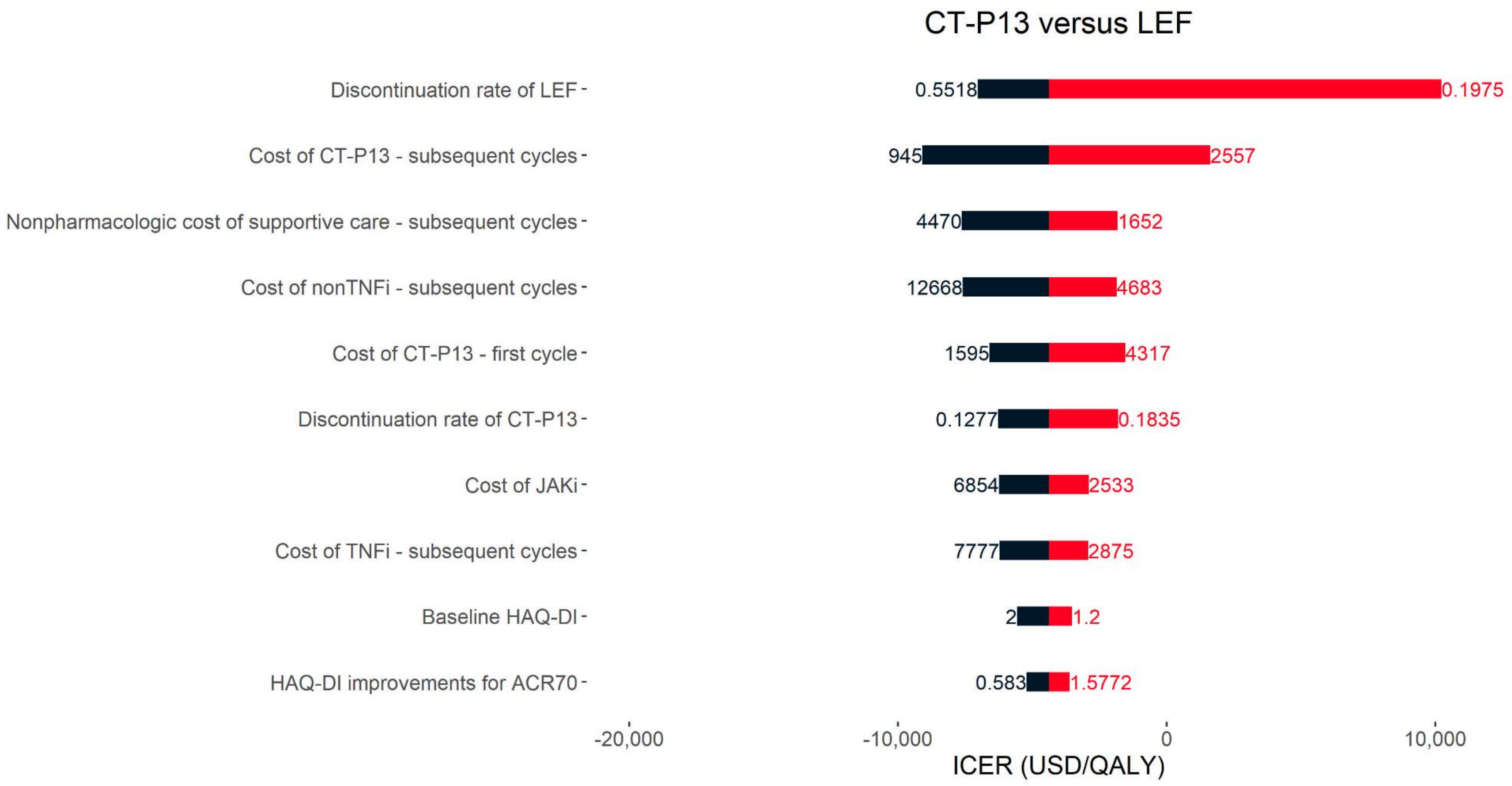

eFigure 2: Deterministic sensitivity analysis comparing CT-P13 against LEF

Tornado diagram representing the incremental cost-effectiveness ratio (ICER) in deterministic sensitivity for CT-P13 versus Leflunomide (LEF). The width of the bars represents the range of the ICER when the parameters changed. TNFi: Tumour Necrosis Factor Inhibitors; JAKi: Janus kinase inhibitor; HAQ-DI: Health Assessment Questionnaire Disability Index; ACR: American College of Rheumatology; QALY: Quality adjusted life year.

© 2024 Peng K et al. *JAMA Network Open.*

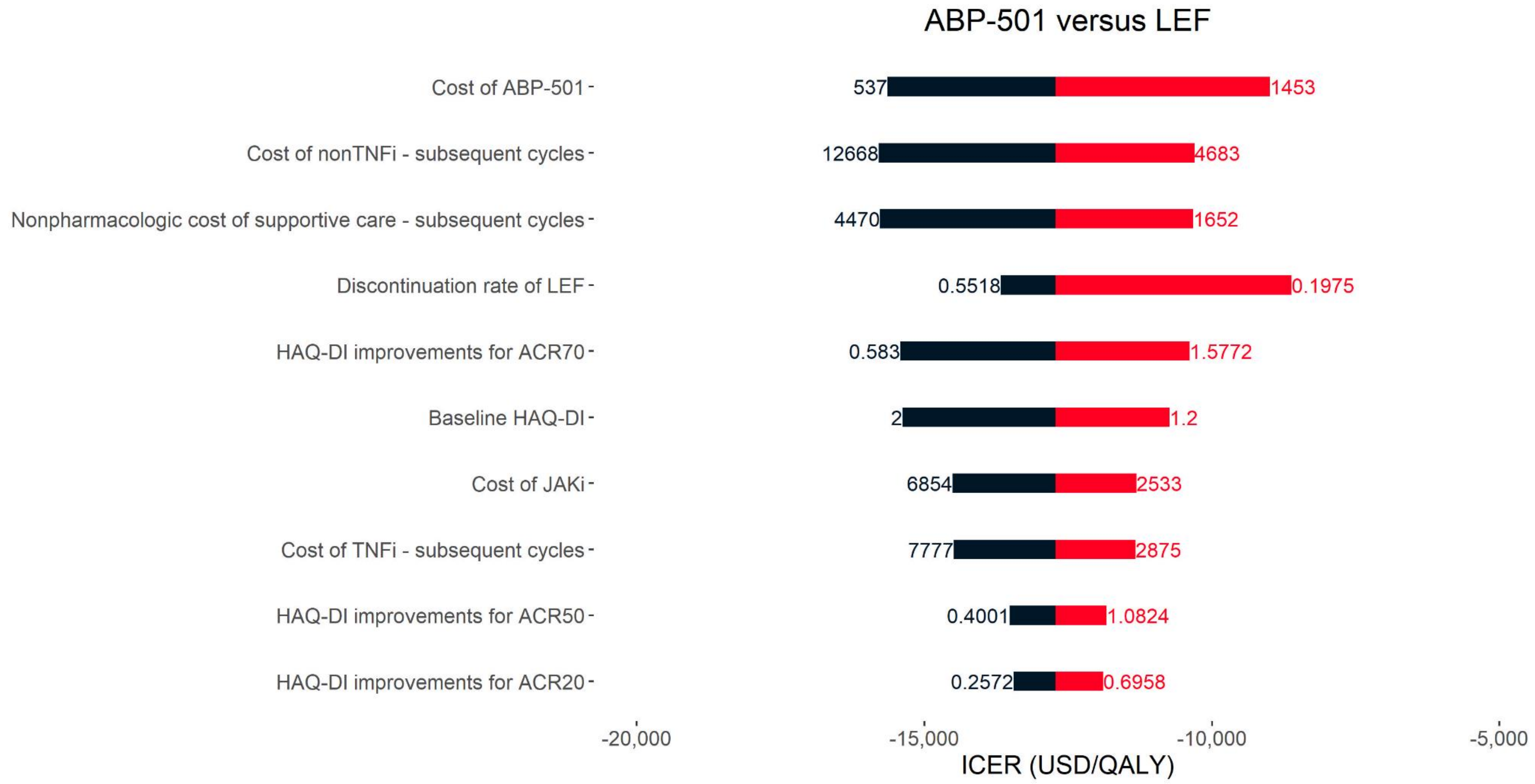

eFigure 3: Deterministic sensitivity analysis comparing ABP-501 against LEF

Tornado diagram representing the incremental cost-effectiveness ratio (ICER) in deterministic sensitivity for ABP-501 versus Leflunomide (LEF). The width of the bars represents the range of the ICER when the parameters changed. TNFi: Tumour Necrosis Factor Inhibitors; JAKi: Janus kinase inhibitor; HAQ-DI: Health Assessment Questionnaire Disability Index; ACR: American College of Rheumatology; QALY: Quality adjusted life year.

© 2024 Peng K et al. *JAMA Network Open.*

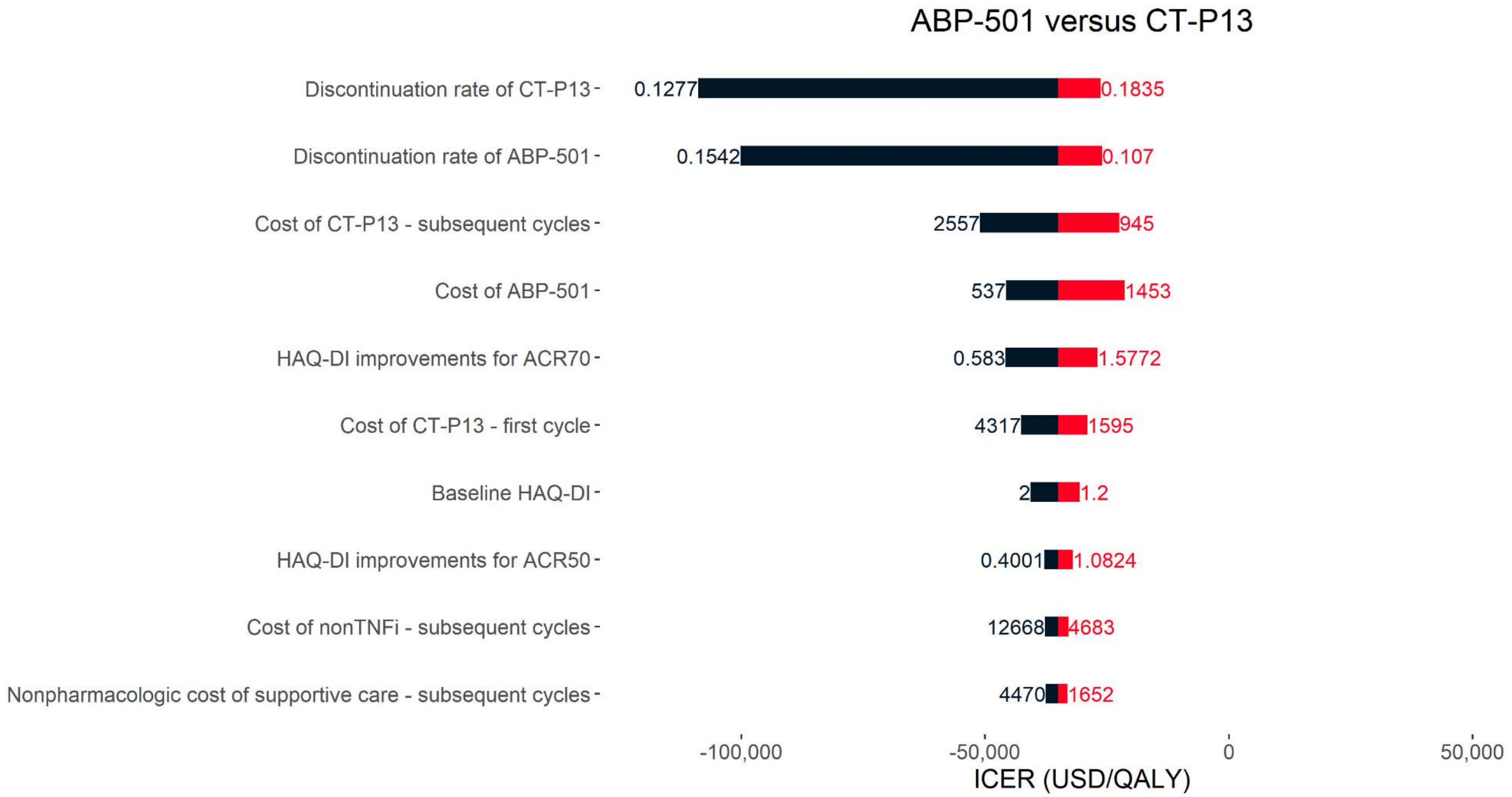

eFigure 4: Deterministic sensitivity analysis comparing ABP-501 against CT-P13

Tornado diagram representing the incremental cost-effectiveness ratio (ICER) in deterministic sensitivity for ABP-501 versus CT-P13. The width of the bars represents the range of the ICER when the parameters changed. TNFi: Tumour Necrosis Factor Inhibitors; JAKi: Janus kinase inhibitor; HAQ-DI: Health Assessment Questionnaire Disability Index; ACR: American College of Rheumatology; QALY: Quality adjusted life year.

© 2024 Peng K et al. *JAMA Network Open.*

eReferences

1. Chen YF, Jobanputra P, Barton P, et al. A systematic review of the effectiveness of adalimumab, etanercept and infliximab for the treatment of rheumatoid arthritis in adults and an economic evaluation of their cost-effectiveness. *Health Technol Assess.* 2006;10(42):iii-iv, xi-xiii, 1-229.
2. Keystone EC, Kavanaugh AF, Sharp JT, et al. Radiographic, clinical, and functional outcomes of treatment with adalimumab (a human anti-tumor necrosis factor monoclonal antibody) in patients with active rheumatoid arthritis receiving concomitant methotrexate therapy: a randomized, placebo-controlled, 52-week trial. *Arthritis Rheum.* 2004;50(5):1400-1411.
3. Yoo DH, Hrycaj P, Miranda P, et al. A randomised, double-blind, parallel-group study to demonstrate equivalence in efficacy and safety of CT-P13 compared with innovator infliximab when coadministered with methotrexate in patients with active rheumatoid arthritis: the PLANETRA study. *Ann Rheum Dis.* 2013;72(10):1613-1620.
4. Keystone EC, Genovese MC, Klareskog L, et al. Golimumab, a human antibody to tumour necrosis factor {alpha} given by monthly subcutaneous injections, in active rheumatoid arthritis despite methotrexate therapy: the GO-FORWARD Study. *Ann Rheum Dis.* 2009;68(6):789-796.
5. Weinblatt ME, Kremer JM, Bankhurst AD, et al. A trial of etanercept, a recombinant tumor necrosis factor receptor:Fc fusion protein, in patients with rheumatoid arthritis receiving methotrexate. *N Engl J Med.* 1999;340(4):253-259.
6. Keystone E, Heijde D, Mason D, Jr., et al. Certolizumab pegol plus methotrexate is significantly more effective than placebo plus methotrexate in active rheumatoid arthritis: findings of a fifty-two-week, phase III, multicenter, randomized, double-blind, placebo-controlled, parallel-group study. *Arthritis Rheum.* 2008;58(11):3319-3329.
7. Bombardieri S, Ruiz AA, Fardellone P, et al. Effectiveness of adalimumab for rheumatoid arthritis in patients with a history of TNF-antagonist therapy in clinical practice. *Rheumatology.* 2007;46(7):1191-1199.
8. Fleischmann R, Goldman JA, Leirisalo-Repo M, et al. Infliximab efficacy in rheumatoid arthritis after an inadequate response to etanercept or adalimumab: results of a target-driven active switch study. *Curr Med Res Opin.* 2014;30(11):2139-2149.
9. Smolen JS, Kay J, Doyle MK, et al. Golimumab in patients with active rheumatoid arthritis after treatment with tumour necrosis factor α inhibitors (GO-AFTER study): a multicentre, randomised, double-blind, placebo-controlled, phase III trial. *The Lancet.* 2009;374(9685):210-221.
10. Bessette L, Khraishi M, Kivitz AJ, et al. Single-Arm Study of Etanercept in Adult Patients with Moderate to Severe Rheumatoid Arthritis Who Failed Adalimumab Treatment. *Rheumatol Ther.* 2017;4(2):391-404.
11. Bessette L, Haraoui B, Chow A, et al. Effectiveness and safety of certolizumab pegol in rheumatoid arthritis patients in Canadian practice: 2-year results from the

© 2024 Peng K et al. *JAMA Network Open.*

observational FαsT-CAN study. *Ther Adv Musculoskelet Dis.* 2019;11:1759720X19831151.

12. Genovese MC, Schiff M, Luggen M, et al. Longterm Safety and Efficacy of Abatacept Through 5 Years of Treatment in Patients with Rheumatoid Arthritis and an Inadequate Response to Tumor Necrosis Factor Inhibitor Therapy. *J Rheumatol.* 2012;39(8):1546.
13. Cohen SB, Emery P, Greenwald MW, et al. Rituximab for rheumatoid arthritis refractory to anti-tumor necrosis factor therapy: Results of a multicenter, randomized, double-blind, placebo-controlled, phase III trial evaluating primary efficacy and safety at twenty-four weeks. *Arthritis Rheum.* 2006;54(9):2793-2806.
14. Fleischmann R, van Adelsberg J, Lin Y, et al. Sarilumab and Nonbiologic Disease-Modifying Antirheumatic Drugs in Patients With Active Rheumatoid Arthritis and Inadequate Response or Intolerance to Tumor Necrosis Factor Inhibitors. *Arthritis Rheumatol.* 2017;69(2):277-290.
15. Strand V, Burmester GR, Ogale S, Devenport J, John A, Emery P. Improvements in health-related quality of life after treatment with tocilizumab in patients with rheumatoid arthritis refractory to tumour necrosis factor inhibitors: results from the 24-week randomized controlled RADIATE study. *Rheumatology.* 2012;51(10):1860-1869.
16. Burmester GR, Blanco R, Charles-Schoeman C, et al. Tofacitinib (CP-690,550) in combination with methotrexate in patients with active rheumatoid arthritis with an inadequate response to tumour necrosis factor inhibitors: a randomised phase 3 trial. *Lancet.* 2013;381(9865):451-460.
17. Genovese MC, Kremer J, Zamani O, et al. Baricitinib in Patients with Refractory Rheumatoid Arthritis. *N Engl J Med.* 2016;374(13):1243-1252.
18. HOSPITAL AUTHORITY. Fees and Charges. *Public Charges – Non-eligible Persons* https://www.ha.org.hk/visitor/ha_visitor_index.asp?Content_ID=10045&Lang=ENG. Accessed Dec 30, 2022.

© 2024 Peng K et al. *JAMA Network Open.*
