# Supplementary material for: Cost-Effectiveness of Biosimilars vs Leflunomide in Patients With Rheumatoid Arthritis
Source: JAMA Netw Open. 2024 Jun 26;7(6):e2418800. doi: 10.1001/jamanetworkopen.2024.18800 (PMC11208978; doi:10.1001/jamanetworkopen.2024.18800)
Supplement: Supplement 2. — Data Sharing Statement [file jamanetwopen-e2418800-s002.pdf]

Data Sharing Statement

Peng. Cost-Effectiveness of Biosimilars vs Leflunomide in Patients With Rheumatoid Arthritis. *JAMA Netw Open*. Published June 26, 2024. doi:10.1001/jamanetworkopen.2024.18800

Data
Data available: No
